# Supplementary material for: Mapping the evolution of fertility support policies in China: A content and instrumental analysis
Source: PLoS One. 2025 Oct 9;20(10):e0332137. doi: 10.1371/journal.pone.0332137 (PMC12510515; doi:10.1371/journal.pone.0332137)
Supplement: S1 Appendix — (ZIP) [file pone.0332137.s001.zip › S1 Appendix. 226 original policy documents/83-国务院关于印发中国21世纪初可持续发展行动纲要的通知(FBM-CLI-2-44947).doc]

国务院关于印发中国21世纪初可持续发展行动纲要的通知

**发布部门：** [国务院](https://www.pkulaw.com/law/chl?Aggs.IssueDepartment=5)

**发文字号：**国发〔2003〕3号

**发布日期：**2003.01.14

**实施日期：**2003.01.14

**时效性：** [现行有效](https://www.pkulaw.com/law/chl?Aggs.TimelinessDic=01)

**效力级别：** [国务院规范性文件](https://www.pkulaw.com/law/chl?Aggs.EffectivenessDic=XC0203)

**法规类别：** [宏观经济体制改革](https://www.pkulaw.com/law/chl?Aggs.Category=02301)

　　　　　　国务院关于印发中国21世纪初可持续发展行动纲要的通知
　　　　　　　　　　　　　　（国发[2003]3号）

各省、自治区、直辖市人民政府，国务院各部委、各直属机构：
　　现将国家计委会同有关部门制订的《中国21世纪初可持续发展行动纲要》印发给你们，请结合本地区、本部门实际，认真贯彻执行。
　　《中国21世纪初可持续发展行动纲要》（以下简称《纲要》）提出了我国可持续发展的目标、重点领域和保障措施，是进一步推进我国可持续发展的重要政策文件。各地区、各部门都应在《纲要》的指导下，认真组织制定可持续发展相关领域的行动规划和计划，并按照综合规划，突出重点，相互协调、充分参与的原则加以实施。要进一步加大宣传力度，增强可持续发展意识，动员社会各方面力量，推进可持续发展。各地区在开展各种类型的可持续发展试点工作过程中，要及时总结经验，逐步推广。国务院有关部门要以高度的责任感和使命感，认真做好综合协调、督促指导、政策研究和信息交流工作，按照与时俱进的要求，积极推进《纲要》的实施。
　　　　　　　　　　　　　　　　　　　　　　　　　　　　二OO三年一月十四日
　　　　　　　　　 中国21世纪初可持续发展行动纲要
　　1992年联合国环境与发展大会后，我国政府率先组织制定了《中国21世纪议程—中国21世纪人口、环境与发展白皮书》，作为指导我国国民经济和社会发展的纲领性文件，开始了我国可持续发展的进程。为了全面推动可持续发展战略的实施，明确21世纪初我国实施可持续发展战略的目标、基本原则、重点领域及保障措施，保证我国国民经济和社会发展第三步战略目标的顺利实现，在总结以往成就和经验的基础上，根据新的形势和可持续发展的新要求，特制定《中国21世纪初可持续发展行动纲要》(以下简称《纲要》)。

　第一部分　成就与问题

　　经过10年的努力，我国实施可持续发展取得了举世瞩目的成就。
　　——经济发展方面。国民经济持续、快速、健康发展，综合国力明显增强，国内生产总值已超过10万亿元，成为发展中国家中吸引外国直接投资最多的国家和世界第6大贸易国，人民物质生活水平和生活质量有了较大幅度的提高，经济增长模式正在由粗放型向集约型转变，经济结构逐步优化。
　　——社会发展方面。人口增长过快的势头得到遏制，科技教育事业取得积极进展，社会保障体系建设、消除贫困、防灾减灾、医疗卫生、缩小地区发展差距等方面都取得了显著成效。
　　——生态建设、环境保护和资源合理开发利用方面。国家用于生态建设、环境治理的投入明显增加，能源消费结构逐步优化，重点江河水域的水污染综合治理得到加强，大气污染防治有所突破，资源综合利用水平明显提高，通过开展退耕还林、还湖、还草工作，生态环境的恢复与重建取得成效。
　　——可持续发展能力建设方面。各地区、各部门已将可持续发展战略纳入了各级各类规划和计划之中，全民可持续发展意识有了明显提高，与可持续发展相关的法律法规相继出台并正在得到不断完善和落实。
　　但是，我国在实施可持续发展战略方面仍面临着许多矛盾和问题。
　　——制约我国可持续发展的突出矛盾主要是：经济快速增长与资源大量消耗、生态破坏之间的矛盾，经济发展水平的提高与社会发展相对滞后之间的矛盾，区域之间经济社会发展不平衡的矛盾，人口众多与资源相对短缺的矛盾，一些现行政策和法规与实施可持续发展战略的实际需求之间的矛盾等。
　　——亟待解决的问题主要有：人口综合素质不高，人口老龄化加快，社会保障体系不健全，城乡就业压力大，经济结构不尽合理，市场经济运行机制不完善，能源结构中清洁能源比重仍然很低，基础设施建设滞后，国民经济信息化程度依然很低，自然资源开发利用中的浪费现象突出，环境污染仍较严重，生态环境恶化的趋势没有得到有效控制，资源管理和环境保护立法与实施还存在不足。
　　随着经济全球化的不断发展，国际社会对可持续发展与共同发展的认识不断深化，行动步伐有所加快。我国应以加入世贸组织为契机，充分发挥社会主义市场经济体制的优越性，进一步发挥政府在组织、协调可持续发展战略中的作用，正确处理好经济全球化与可持续发展的关系，抓住2002年联合国可持续发展世界首脑会议成功召开的契机，进一步积极参与国际合作，维护国家的根本利益，保障我国的国家经济安全和生态环境安全，促进我国可持续发展战略的顺利实施。

　第二部分　指导思想、目标与原则

　　**一、**指导思想
　　我国实施可持续发展战略的指导思想是：坚持以人为本，以人与自然和谐为主线，以经济发展为核心，以提高人民群众生活质量为根本出发点，以科技和体制创新为突破口，坚持不懈地全面推进经济社会与人口、资源和生态环境的协调，不断提高我国的综合国力和竞争力，为实现第三步战略目标奠定坚实的基础。

　　**二、**发展目标
　　我国21世纪初可持续发展的总体目标是：可持续发展能力不断增强，经济结构调整取得显著成效，人口总量得到有效控制，生态环境明显改善，资源利用率显著提高，促进人与自然的和谐，推动整个社会走上生产发展、生活富裕、生态良好的文明发展道路。
　　——通过国民经济结构战略性调整，完成从“高消耗、高污染、低效益”向“低消耗、低污染、高效益”转变。促进产业结构优化升级，减轻资源环境压力，改变区域发展不平衡，缩小城乡差别。
　　——继续大力推进扶贫开发，进一步改善贫困地区的基本生产、生活条件，加强基础设施建设，改善生态环境，逐步改变贫困地区经济、社会、文化的落后状况，提高贫困人口的生活质量和综合素质，巩固扶贫成果，尽快使尚未脱贫的农村人口解决温饱问题，并逐步过上小康生活。
　　——严格控制人口增长，全面提高人口素质，建立完善的优生优育体系和社会保障体系，基本实现人人享有社会保障的目标；社会就业比较充分；公共服务水平大幅度提高；防灾减灾能力全面提高，灾害损失明显降低。加强职业技能培训，提高劳动者素质，建立健全国家职业资格证书制度。到2010年，全国人口数量控制在14亿以内，年平均自然增长率控制在9％。以内。全国普及九年义务教育的人口覆盖率进一步提高，初中阶段毛入学率超过95％，高等教育毛入学率达到20％左右，青壮年非文盲率保持在95％以上。
　　——合理开发和集约高效利用资源，不断提高资源承载能力，建成资源可持续利用的保障体系和重要资源战略储备安全体系。
　　——全国大部分地区环境质量明显改善，基本遏制生态恶化的趋势，重点地区的生态功能和生物多样性得到基本恢复，农田污染状况得到根本改善。到2010年，森林覆盖率达到20．3％，治理“三化” (退化、沙化、碱化)草地3300万公顷，新增治理水土流失面积5000万公顷，二氧化硫、工业固体废物等主要污染物排放总量比前5年下降10％，设市城市污水处理率达到60％以上。
　　——形成健全的可持续发展法律、法规体系；完善可持续发展的信息共享和决策咨询服务体系；全面提高政府的科学决策和综合协调能力；大幅度提高社会公众参与可持续发展的程度；参与国际社会可持续发展领域合作的能力明显提高。

　　**三、**基本原则
　　——持续发展，重视协调的原则。以经济建设为中心，在推进经济发展的过程中，促进人与自然的和谐，重视解决人口、资源和环境问题，坚持经济、社会与生态环境的持续协调发展。
　　——科教兴国，不断创新的原则。充分发挥科技作为第一生产力和教育的先导性、全局性和基础性作用，加快科技创新步伐，大力发展各类教育，促进可持续发展战略与科教兴国战略的紧密结合。
　　——政府调控，市场调节的原则。充分发挥政府、企业、社会组织和公众四方面的积极性，政府要加大投入，强化监管，发挥主导作用，提供良好的政策环境和公共服务，充分运用市场机制，调动企业、社会组织和公众参与可持续发展。
　　——积极参与，广泛合作的原则。加强对外开放与国际合作，参与经济全球化，利用国际、国内两个市场和两种资源，在更大空间范围内推进可持续发展。
　　——重点突破，全面推进的原则。统筹规划，突出重点，分步实施；集中人力、物力和财力，选择重点领域和重点区域，进行突破，在此基础上，全面推进可持续发展战略的实施。

　第三部分　重点领域

　　**一、**经济发展
　　按照“在发展中调整，在调整中发展”的动态调整原则，通过调整产业结构、区域结构和城乡结构，积极参与全球经济一体化，全方位逐步推进国民经济的战略性调整，初步形成资源消耗低、环境污染少的可持续发展国民经济体系。
　　——产业结构调整。对农业和农村经济结构进行战略性调整，提高土地和水资源的利用率，减少对环境的污染和对生态环境的破坏；调整种植业、养殖业内部结构，优化农业生产区域布局，大力推进农业产业化经营，推进乡镇企业技术进步和体制创新。对工业进行改组改造和结构优化升级，减少产业发展对资源环境造成的压力，用高新技术和先进适用技术改造提升传统产业，有重点地改造一批骨干企业和发展一批高技术工程。大力发展服务业，提高供给能力和服务水平，满足人民生活质量日益增长的需要，发展以住宅为重点的房地产业，加强旅游基础设施和配套设施建设，优化配置和充实社区服务设施，壮大社区服务业，改造提升传统流通业、运输业和邮政服务业；发展信息产业，实施信息化战略，推进政务、金融、外贸、广播电视、教育、科技、医疗卫生、社会保障和公用事业等重点领域信息化进程。加强基础设施建设，加强以大江大河治理为重点的防洪工程建设，加快南水北调等水资源宏观配置工程，加快重点公路国道主干网建设，建设改造主要铁路通道。
　　——区域发展与消除贫困。调整区域结构，减缓区域发展不平衡，通过扶贫和推进西部大开发战略，加快西部地区的水利、交通、能源、通信、广播电视和城市基础设施建设，加快发展特色经济，提高资源利用效率，减少污染物排放；逐步消除绝对贫困和减少相对贫困，继续实行开发式扶贫，实行政府主导，社会各界参与扶贫开发，采取社会、经济、生态环境综合配套的扶贫措施，改善贫困地区生产、生活、医疗卫生等基本条件；积极稳妥地开展生态移民，减轻生态恶化地区的压力，促进生态保护和恢复。
　　——城镇化与小城镇建设。加强城镇体系规划，积极发展中小城市，完善区域性中心城市的功能，发挥大城市的辐射带动作用，有重点地发展小城镇。适时、科学、稳妥地调整城镇行政区划设置，构建适应我国城镇可持续发展的体制框架、政策框架和规划体系，积极稳妥地推进城镇化进程，完善城镇社会经济综合发展规划，分类指导不同类型的城镇发展。发展城乡一体化的劳动力、资金等要素市场，加快农村人口向城镇转移的步伐。把引导农村中小企业合理集聚、完善农村市场体系、发展农业产业化经营和社会化服务等与小城镇建设结合起来，通过繁荣小城镇经济，提高小城镇对农村人口的吸纳能力。加强城镇基础设施建设，提高城镇的就业容量，健全城镇居住、公共服务和社会服务等功能；加强城市社区管理，建设有序、文明、祥和的新型社区，创造良好的人居环境，加强城镇综合治理，改善城镇环境，形成各’具特色的城镇风格，全面提高城镇管理水平。
　　——积极应对经济全球化。优化出口商品结构，完善进口商品管理，扩大机电产品和高新技术产品的出口，限制和禁止影响人类健康、安全、破坏环境的商品和技术进出口；逐步提高进出口商品的环境质量标准，建立健全保护人类健康、动植物健康和环境的管理体制；大力发展国际经贸，吸引外资投向我国鼓励发展的领域和地区，鼓励有实力的国内企业向海外发展；提高产业国际竞争力，将传统的资源密集型产业调整为劳动密集型、技术密集型和资本密集型相结合的产业；积极参与多边贸易、环境等规则制定，与国际社会共同努力，反对利用环境、人权等问题制造新的贸易壁垒，切实保障可持续发展战略的顺利实施。

　　**二、**社会发展
　　建立完善的人口综合管理与优生优育体系，稳定低生育水平，控制人口总量，提高人口素质。建立与经济发展水平相适应的医疗卫生体系、劳动就业体系和社会保障体系。大幅度提高公共服务水平。建立健全灾害监测预报、应急救助体系，全面提高防灾减灾能力。
　　——加强人口综合管理。坚持计划生育基本国策，稳定现行生育政策，加快我国有关人口方面的法律法规体系建设；切实加强计划生育基层基础工作，农村抓村组，城市抓社区，落实企事业单位计划生育法人责任制，加强计划生育技术服务网络建设，提高干部队伍管理和服务水平；加强社会主义新型生育观念和生育文化宣传教育，加强计划生育利益导向机制建设，加强与计划生育相关的社会保障制度建设；采取多种综合措施，鼓励家庭实行计划生育，加强计划生育人群权益保护；加强对流动人口的综合管理；高质量、高水平地普及九年义务教育，搞好学前教育，在城市和发达地区普及高中阶段教育，加强高等教育，发展继续教育，提高人口素质。
　　——完善社会保障体系。建立独立于企业事业单位之外，资金来源多元化、保障制度规范化、管理服务社会化的社会保障体系。调整和完善城镇职工基本养老保险制度；稳步推动国有企业下岗职工基本生活保障向失业保险并轨，健全失业保险制度；努力扩大基本医疗保险覆盖面，稳步推进工伤保脸和生育保险；加强和完善以城市居民最低生活保障制度为基础的社会救济制度，加快社会福稠特别是社区老年福利事业的发展，在有条件的地区探索建立农村养老、医疗保险和最低生活保障制度；实现社会保障管理和服务的社会化；加强社会保障资金的筹集和管理；加快社会保障立法步伐；加快牡会保障信息系统建设；实行积极的就业政策，千方百计扩大就业，广开就业门路，发展劳动密集型产业；引导全社会转变就业观念，推行灵活多样的就业形式，鼓励自谋职业和自主创业；完善就业服务体系，加强职业技能培训，提高劳动者素质。
　　——发展卫生事业。建立健全卫生法律法规体系、监督执法体系。继续深化医疗卫生管理体制改革，完善政府调控下的各项医疗卫生管理政策；优化卫生资源配置，合理布局，逐步形成以社区卫生服务为基础、分工合理、方便、快捷的新型城镇卫生服务体系；加强农村卫生事业建设，建立适应农村社会经济发展要求，具有预防保健和基本医疗功能的农村卫生服务体系，基本实现农民人人享有初级卫生保健目标；提高对突发事件、紧急疫情的迅速反应和处理能力，加强对重大疾病的预防与有效控制；切实加强妇女儿童的预防保健工作，提高儿童全程免疫接种率，全面提高妇女儿童的保健水平；加强社会养老保障建设，完善老年医疗、康复服务以及医院、社区卫生服务与家庭护理服务体系；加强职业病的防治，保护从业人员健康；全面普及卫生知识，提倡健康的生活习惯与生活方式。
　　——加强灾害综合管理。进一步完善灾害管理法律法规，增强全民防灾减灾意识；耐定防灾减灾规划和应急方案；建立和完善主要自然灾害以及重大事故的监测、预报预警系统，全面提升和整合信息处理能力，提高预报的时效性和准确性；加强部门协作与配合，建立完善的灾害处理应急指挥系统和减灾救灾综合协调机制，加强防灾减灾工程建设、救援物资和设备储备，形成一支快速反应的救灾力量，提高紧急救援能力，减少灾害造成的人员伤亡和经济损失；推进经常性社会捐助工作网络建设和制度建设，逐步形成良好的社会化灾害救助机制。

　　**三、**资源优化配置、合理利用与保护
　　合理使用、节约和保护资源，提高资源利用率和综合利用水平。建立重要资源安全供应体系和战略资源储备制度，最大限度地保证国民经济建设对资源的需要。
　　——水资源优化配置、合理利用、有效保护与安全供给。健全水资源开发、管理与保护的法律法规体系，实施更严格的水资源管理政策；实施水资源流域管理与行政区域管理相结合的管理体制；明确水权，实施流域与区域水资源总量的分配制度，合理调配生活、生产和生态用水；实施国民经济和社会发展规划、城市总体规划、重大建设项目水资源论证制度，促进水资源利用与人口、环境、经济社会协调发展；建立合理的价格机制和激励机制，实施计划用水与定额用水柑结合的综合管理措施，推行用水审计，促进水资源的合理利用；鼓励发展节水农业、节水工业，建设节水型城市和节水型社会，全面节约用水，提高水的利用率；实施水功能区划管理制度，有效保护水资源；实施南水北调工程，改善水资源的宏观布局；大力提倡污水再生利用等非传统水资源的开发利用；在沿海地区积极开发利用海水资源，推广应用海水淡化技术，补充陆地淡水不足。
　　——土地合理利用。贯彻执行十分珍惜、合理利用土地和切实保护耕地的基本国策，坚持实行世界上最严格的土地管理制度；加强土地资源调查、评价和监测，科学编制和严格实施土地利用总体规划；加强耕地保护和基本农田建设，防止耕地质量退化，确保国家粮食安全；加强林地保护和森林资源建设，确保国土生态安全；合理调整土地利用结构与布局，提高土地利用效率；加强建设用地管理，控制建设用地规模，促进农民居住向城镇集中、工业向工业园区集中，保障经济建设必需的土地；积极开展土地整理和复垦，适度开发土地后备资源；加强土地资产管理，深化土地使用制度改革，大力推进土地使用权市场建设?完善地价管理制度和土地税费体系，引导集约高效利用土地资源；完善土地产权制度，保障农户承包经营权；改革征地制度；深入开展土地科技的研究与应用，扩大土地遥感监测，逐步完善土地利用管理信息系统；加强土地法制建设，完善土地管理法律法规，健全土地执法体制，加大土地执法力度。
　　——改善能源结构，提高能源效率。大力发展天然气、水电、可再生能源、新能源等清洁能源，发展清洁燃料公共汽车和电动公共汽车，积极利用国外油气资源，努力降低煤炭在一次能源消费中的比重；大力发展清洁利用煤炭和热电联产集中供热技术，强化能源节约，提高能源利用效率，减少环境污染；在适宜地区大力发展沼气、节能灶、太阳能、风能等，改善农村能源结构；实施“西气东输”、“西电东送”等重大工程，改善能源布局。
　　——森林资源的可持续利用。加大森林资源保护、管理、监督和执法力度，制止乱砍滥伐林木、毁林开垦等行为，提高全民保护森林资源的意识；积极落实森林资源管护经营责任制，明确“责、权、利”关系，理顺森林资源管理体制；深化林业的分类经营改革；调整和完善不适应的林业政策，切实减轻林业税费负担；提高科技含量，以高新技术改造传统产业和淘汰落后生产方式，加快发展速生丰产用材林基地和林业产业；加大生态环境建设力度，全面实施天然林资源保护等林业重点工程建设。
　　——草地资源的可持续利用。加强草原管理机构建设，强化管理职能，加大执法力度；积极落实草原承包制，明确草原使用的“责、权、利”关系；提高科技含量，改变草原资源利用方式，变传统的粗放数量型为质量效益型；加大以人工种草、飞播种草、围栏封育、草场改良、划区轮牧和草地鼠虫害防治等为主要内容的天然草原保护建设实施力度，防止超载过牧，强化“三化”草地治理，恢复天然草场植被。
　　——矿产资源的可持续利用。进一步健全矿产资源法律法规体系；科学编制和严格实施矿产资源规划，加强对矿产资源开发利用的宏观调控，促进矿产资源勘查和开发利用的合理布局。进一步加强矿产资源调查评价和勘查工作，提高矿产资源保证程度；对战略性矿产资源实行保护性开采。健全矿产资源有偿使用制度，依靠科技进步和科学管理，促进矿产资源利用结构的调整和优化，(提高资源利用效率。充分利用国内外资金、资源和市场，建立大型矿产资源基地和海外矿产资源基地。加强矿山生态环境恢复治理和保护。
　　——海洋资源的可持续利用。制定合理利用和保护海洋的发展规划；严格执行海洋功能区划，强化海域使用管理，加强海域使用审批，全面推行海域有偿使用制度；加强海洋监测、执法管理系统建设；开展全国性海洋生态环境调查与研究；大力发展海洋高新技术，积极开发利用深海和大洋资源。
　　——气候资源的可持续利用。进一步增强全民的气候资源意识；建立和健全气候资源开发利用与保护的法律法规体系；制定气候资源合理开发利用与保护规划；及时修订、更新气候资源区划；采用先进的计算机信息处理技术和遥感技术，加强对气候资源的监测与评估；建立气候资源合理开发利用的试验示范基地。重点做好农业气候资源、风能、太阳能的监测、区划、规划和试验示范工作。
　　——矿产资源战略储备。建立战略矿产资源储备制度，完善相关经济政策和管理体制；建立战略矿产资源安全供应的预警系统，在大城市实行多水源供给的水资源战略储备制度；采用国家储备与社会储备相结合的方式，实施石油等重要矿产资源战略储备。

　　**四、**生态保护和建设
　　建立科学、完善的生态环境监测、管理体系，形成类型齐全、分布合理、面积适宜的自然保护区，建立沙漠化防治体系，强化重点水土流失区的治理，改善农业生态环境，加强城市绿地建设，逐步改善生态环境质量。
　　——生态环境监测及安全评价。建立完善的生态环境监测与安全评估技术和标准体系，形成国家级、区域级、保护区等多层次的生态环境监测体系；采用遥感和地面监测等现代技术手段对森林、草地、湿地、农田、自然保护区、沙漠、水土保持、农业生态环境、生物多样性、大型生态建设工程、重点资源开发区及土地利用变化等进行有效监测与管理，对严重突发污染事故和海上赤潮、石油污染、沙尘暴等灾害进行应急跟踪监测；建立生态环境安全评价及预警预报系统。
　　——建设林业重点生态工程。重点保护长江上游、黄河中上游和东北国有林区天然林资源，治理水。土流失，减少风沙危害，加强生物多样性保护，建立速生丰产林基地，逐步满足人们对生态环境和林副产品的需求。加快实施天然林保护，退耕还林，京津风沙源治理，“三北”和长江中下游地区等重点防护林建设、野生动植物及自然保护区建设、重点地区速生丰产用材林基地建设六大林业生态工程。
　　——建立自然保护区。加强现有森林生态系统、珍稀野生动物、荒漠生态系统、内陆湿地和水域生态系统等类型自然保护区建设，强化现有草原与草甸生态系统、海洋和海岸生态系统、野生植物、地质遗迹、古生物遗迹等类型自然保护区的建设；在长江、黄河等大江大河源头区域及青藏高原的重要天然湿地，西南、东北以及西北荒漠地区等生物多样性丰富、原生生态系统保存较好且生态敏感区域以及珍稀濒危物种的栖息地，有计划地建立一批质量高、有实效的自然保护区。合理空间布局，加强生物走廊带建设。
　　——建立生态功能保护区。加强现有生态功能保护区的建设和管理；在江河源区，长江、黄河和松花江等流域重要湿地(湖泊)，塔里木河、黑河等内陆河流域，南方红壤丘陵区、黄土高原、北方土石山区，农牧交错区、干旱草原地区，近海重要渔业水域建立生态功能保护区；调整生态功能保护区内的产业结构，发展生态“友好型”的产业，最大限度地减轻人为活动对生态系统的影响；坚持“封育为主，宜治则治，宜荒则荒”的原则，尽快恢复与重建生态功能。
　　——防治土地沙化。制定适合土地沙化地区经济发展的经营机制和政策，研究、推广防治土地沙化的适应耕作制度；形成防、治、用有机结合的土地沙化防治体系；干旱沙漠边缘及绿洲类型区以保护现有植被为主，在绿洲外围建立综合防护体系；半干旱沙地类型区主要是保护和恢复林草植被；高原高寒沙化土地类型区主要是在做好现有植被保护的前提下，对人类经济活动集中地区的沙化土地进行治理；黄淮海平原半湿润、湿润沙地类型区应全面治理沙化土地并适度开发利用，南方湿润沙地类型区应对沙化土地进行综合治理和开发。对不具备治理条件和不宜开发利用的连片沙化土地，采取措施，严禁开拔。
　　——加强水土保持。完善水土保持政策，落实国家对退耕还林、还草的各项政策，加强基本农田和草原水利建设；坚持水资源保护与开发相结合，水土流失治理与群众脱贫致富、发展地方经济相结合的原则，实施以大流域为骨干、以小流域为单元的综合治理，防止大规模开发建设过程中造成新的人为水土流失；建立工程措施、生物措施和耕作措施相结合的综合防治体系；研究、开发和推广水土保持实用技术，加强国际合作与交流，引进和推广先进技术、优良品种、管理方法和手段。
　　——发展生态农业。加大对农业野生生物资源保护的监督管理力度，抢救性收集重点地区农业野生生物资源，建立农业野生生物原生地保护示范区或保护点；大力开展保护性耕作、继续开展旱作农业示范县和生态农业示范县建设；制订有效的政策及管理机制，研究、推广生态农业关键技术和模式，鼓励农民采用清洁生产技术，合理使用化肥、农药，开展农业废弃物综合利用，建立可持续发展的农业生态系统；健全无公害农产品法律法规、标准、检测体系，完善农业生态环境检测、评价及预警系统；强化农产品生产基地及产品安全管理，创建各类特色的无公害农产品生产示范基地及品牌。
　　——加强风景名胜区保护。按照“严格保护、统一管理、合理开发、永续利用”的原则，编制风景名胜区规划，并严格实施。风景名胜区规划中要划定核心保护区(包括生态保护区、自然景观区和史迹保护区)保护范围，制定专项保护规划，确定保护重点和保护措施。核心保护区禁止与资源保护无关的工程建设。风景名胜区规划要与当地土地利用总体规划协调一致。
　　——重视城市生态环境建设。合理规划城市建设用地，建立并严格实施城市“绿线”管制制度。按现代化城市的标准，确保一定比例的公共绿地和较大面积的城市周边生态保护区域。加大城市绿化建设力度，提高城市大气环境质量。大力推动园林城市创建活动，减轻“城市热岛效应”。加强城市建设项目环境保护及市容环境管理，减少扬尘和噪音。

　　**五、**环境保护和污染防治
　　实施污染物排放总量控制，开展流域水质污染防治，强化重点城市大气污染防治工作，加强重点海域的环境综合整治。加强环境保护法规建设和监督执法，修改完善环境保护技术标准，大力推进清洁生产和环保产业发展。积极参与区域和全球环境合作，在改善我国环境质量的同时，为保护全球环境作出贡献。
　　——流域水污染防治。加大重点河流和湖泊水污染防治力度，加强饮用水源地保护、富营养化湖泊治理、面源污染控制；推行清洁生产，进一步减少污染物排放量；实行流域污染物排放总量控制，提高污水处理率，在一些行业推行污水零排放；继续加大城市污水和垃圾处理设施的建设力度；优化产业、产品结构，发展环保高技术；加快研发和推广适合国情的重污染行业污染治理技术工艺，提升我国整体产业水平和水污染治理水平。
　　——海洋污染防治。完善全国海洋环境监测网络，强化海洋污染及生态环境监测；逐步减少陆源污染物向海排放和各种海洋生产、开发活动对海洋造成的污染，实施污染物人海总量控制制度；开展重点海域的环境综合整治，加大海岸带生态环境保护与建设力度。
　　——大气污染防治。控制致酸物质、有毒有害工业气体排放；防治酸雨、可吸入颗粒物、光化学烟雾和室内空气污染。
　　——城市交通管理。整合城市交通结构，优先发展公共交通，特大城市要注重发展轨道交通，建立公共交通优先的路网系统，控制城市机动车尾气污染和噪声污染。
　　——固体废物污染防治。逐步实行垃圾分类收集，实现垃圾的无害化、减量化、资源化，提高垃圾无害化处理率和综合利用率；进一步提高固体废弃物中可利用物质的综合利用率，加强矿山环境保护和生态恢复治理；加强危险废物的安全处置。
　　——环保产业发展。规范环保产业市场，优化环保产业结构，通过推进污染治理市场化、企业化、产业化，构筑面向市场的环保技术服务体系和良好的市场运行机制；制定引导环保产业发展的配套政策，加强环境保护关键技术和工艺设备的研究开发，提高高效实用环保设备的生产能力，促进重大环保设备的成套化、系列化和标准化；通过环境标准、技术政策、示范工程和重点实用技术等引导环保产业发展；实施ISO14000环境管理体系、环境标志产品认证制度，大力加强环保中介、环保技术和工程服务。

　　**六、**能力建设
　　建立完善人口、资源和环境的法律制度，加强执法力度，充分利用各种宣传教育媒体，全面提高全民可持续发展意识，建立可持续发展指标体系与监测评价系统，建立面向政府咨询、社会大众、科学研究的信息共享体系。
　　——可持续发展的立法与实施。加强可持续发展立法，完善有关法律制度；以可持续发展为原则，制定和完善人口；资源、生态环境、自然灾害防治以及信息资源共享和利用等方面的法规；根据市场经济运行规律和世贸组织的规则，修订相应的法规。建立健全可持续发展的法律实施保障体系，严格依法行政，加强执法监督，切实保证可持续发展的各项法律制度得以实施。
　　——可持续发展指标体系与监测评价。逐步建立符合我国国情、多层次的可持续发展指标体系和监测评价系统，开展国家和地区的可持续发展水平监测评价，定期发布监测评价报告。
　　——可持续发展的信息共享。建立公共基础数据、人口、社会经济、资源、生态、环境和灾害等信息库，实现基于高速网络基础上的、面向社会各界的、具有数据分析与处理能力的信息共享和信息服务体系；建立适应于政府决策的信息共享网络。

　第四部分　保障措施

　　面对新世纪的国际国内环境，为了实现本《纲要》中提出的各项目标，必须采取行政、经济、科技、法律等手段，从加强部门协调、拓宽融资渠道、依靠科教支撑、健全法规制度等方面采取切实有效的保障措施，按照“三个代表”重要思想的要求，以高度的责任感和使命感，切实推进可持续发展战略的顺利实施。
　　**一、**运用行政手段，提高可持续发展的综合决策水平
　　——加强领导，抓好管理。各级政府都要认真贯彻可持续发展战略，将《纲要》所确定的有关人口资源环境等方面的任务纳入日常工作议程。继续坚持和认真落实人口资源环境工作党政一把手亲自抓、负总责的制度，逐级建立和完善严格的责任制。由政府负责，协调部门制定政策、明确任务，落实责任、督促检查，做到责任、措施和投入“三到位”。不断提高政府统筹规划和政策协调以及规范管理的能力和水平。
　　——强化监督，狠抓落实。结合完善规范、科学的公务员政绩考核和奖惩制度，使各级政府主要领导关心和重视人口、资源和环境的可持续发展。各级政府应当确保将可持续发展工作纳入相关的战略、规划和计划，并且贯穿到计划实施的全过程。逐步将领导干部实施可持续发展战略的评估结果和最后验收结果作为定量考核和评估其工作实绩的主要依据。
　　——统一协调，综合决策。建立和完善人口资源环境与经济社会的统一协调机制与综合决策激励机制；制定有利于综合考核地区和企业可持续发展水平的指标体系，试行将资源环境成本纳入国民经济核算体系；试行重大项目和重大决策的可持续发展影响评价制度，建立公众参与综合决策的渠道，提高政府推进可持续发展的公共服务水平。

　　**二、**运用经济手段，建立有利于可持续发展的投入机制
　　——建立有利于可持续发展的相对稳定的资金投入筹措机制。逐步加大各级政府在可持续发展方面的必要投入，引导非国有资金甚至国外资金投入可持续发展领域，大幅度提高实施可持续发展战略的资金保障程度和稳定性，提高对投入可持续发展资金使用的管理水平和投入资金的经济效益。
　　——建立有利于引导各类利益主体参与可持续发展的价格调节机制。通过价格调节，引导各类相关利益主体的法人强化节约资源，严格保护城乡生态环境，真正发挥价格机制在资源的市场供求和可持续利用等方面的调节功能。

　　**三、**运用科教手段，为推进可持续发展提供强有力的支撑
　　——深化科技体制改革，建设国家创新体系。通过机制创新和管理创新，逐步形成既顺应市场经济要求，又符合科技发展自身规律的科技体制。优化科技资源配置，进一步加强与可持续发展相关的科学研究体系、技术开发体系、科技服务体系建设，调动科技人员从事可持续发展领域研究和产业化的积极性、创造性，促进实现人口资源环境的可持续发展。
　　——依靠科技，大力开发、推广和应用先进适用的生态“友好型”实用技术。集中力量研究开发一批对可持续发展有重大影响的关键技术，提高可持续发展技术水平和能力；在加快企业结构调整的过程中，推行清洁生产，并与企业技术进步、节能降耗、资源综合利用和加强企业管理结合起来，加快各类企业环境污染由末端治理向全过程控制的根本转变。加快建立和完善以开展清洁生产为重点的工业污染综合防治模式，鼓励和支持企业采用高新技术，使用新型能源，走低能耗、物耗、少排污的清洁生产发展道路；选择具备条件的地区或企业，扶持建立若干生态和环保研发中心，组织开展技术和装备攻关，提高环保设备成套化、系列化水平。
　　——积极发展各级各类教育，提高全民可持续发展意识。强化人力资源开发，提高公众参与可持续发展的科学文化素质。在基础教育以及高等教育教材中增加关于可持续发展的内容，在中小学开设“科学”课程，在部分高等学校建立一批可持续发展的示范园(区)。政府有关部门要在经费投入和工作安排上加大面向全社会宣传、普及、推广、应用等软环境建设的力度。科研机构定期向社会开放。利用大众传媒和网络广泛开展国民素质教育和科学普及。加快培育一大批熟悉优生优育、生态环境保护、资源节约、绿色消费等方面基本知识和技能的科研人员、公务员和志愿者。积极鼓励与支持社会组织和民间团体参与促进可持续发展的各项活动。

　　**四、**运用法律手段，提高实施可持续发展战略的法制化水平
　　——继续加强可持续发展方面的立法工作。研究、制定一些新的法律法规，加快修改完善现有法律法规，形成基本完善的可持续发展法律制度。各地区要按照国家法律法规，根据当地实际情况，制定实施一些地方性法规，以促进发展各具特色的区域性可持续发展模式和道路。
　　——做好相应的配套制度建设和标准制定工作。建立健全有关可持续发展的各项管理制度，包括现行的各项环境管理制度、自然资源权属管理制度、有偿使用制度和使用权(产权)流转制度、流动人口综合管理制度等，逐步形成具有中国特’色社会主义的人口资源环境工作管理制度体系；
　　——大力提高全社会的公共监督和法制化管理水平。加强执法队伍建设，加大执法力度，注意发挥新闻单位、社会中介组织的监督作用，切实保障各级政府和执法部门依法行使管理职能；注重人口资源环境有关法律法规知识的宣传、普及和教育，尽快提高社会大众的可持续发展法律意识和法制观念。

　　**五、**运用示范手段，做好重点区域和领域的试点示范工作
　　加强可持续发展的试点示范工作；总结经验，逐步推进可持续发展战略的实施。通过开展试点示范工作，建立一批可持续发展基地，形成集技术、管理、政策、机制于一体的综合示范；围绕可持续发展领域的重大问题，开展政策、战略和关键技术研究。通过典型区域和领域的试点示范，解决地方普遍遇到的资源利用、生态环境保护、结构调整和经济增长方式转变等方面的问题，形成不同类型的可持续发展模式，并向周围其他地区辐射推广。

　　**六、**加强国际合作，为可持续发展创造良好的国际环境
　　积极参与全球环境合作，就参与各类环境条约问题制定对策，认真履行参加的各类国际条约，贯彻落实可持续发展世界首脑会议等相关国际会议达成的决议和决定；重视并积极推动双边和多边国际合作，利用各种渠道促进国内环境保护，维护我国利益，为可持续发展创造良好的国际环境；适应经济全球化和我国加入世贸组织的新形势，逐步建立和完善以绿色产品、技术、服务为主导的投资贸易政策体系，充分利用国际国内两种资源和两个市场，促进我国可持续发展战略的顺利实施。

© 北大法宝：（[www.pkulaw.com](https://www.pkulaw.com/)) 专业提供法律信息、法学知识和法律软件领域各类解决方案。北大法宝为您提供丰富的参考资料，正式引用法规条文时请与标准文本核对。
欢迎查看所有[产品和服务](https://www.pkulaw.com/helps/2.html)。法宝快讯：[如何快速找到您需要的检索结果？](https://www.pkulaw.com/helps/70.html)[法宝](https://www.pkulaw.com/helps/69.html)V6有何新特色？

扫描二维码阅读原文

**原文链接：**<https://www.pkulaw.com/chl/452751e4bffa9537bdfb.html>
